# Temporal Alcohol Availability Predicts First-Time Drunk Driving, but Not Repeat Offending

**DOI:** 10.1371/journal.pone.0071169

**Published:** 2013-08-07

**Authors:** Timothy P. Schofield, Thomas F. Denson

**Affiliations:** School of Psychology, University of New South Wales, Sydney, Australia; California Pacific Medicial Center Research Institute, United States of America

## Abstract

Alcohol availability has been linked to drunk driving, but research has not examined whether this relationship is the same for first-time and repeat offenses. We examined the relationship between the business hours of alcohol outlets licensed to serve alcohol for on-premises consumption and misdemeanor-level (first offense) and felony-level drunk driving (repeat offense) charges in New York State in 2009. Longer outlet business hours were associated with more misdemeanor drunk driving charges, but were not associated with felony drunk driving charges. The per capita density of on-premises alcohol outlets did not affect misdemeanor or felony drunk driving charges. The results suggest that temporal alcohol availability may be an impelling factor for first-time drunk driving, but other factors likely influence repeat drunk driving behaviors.

## Introduction

A staggering 112 million instances of driving while intoxicated (DWI) were estimated to have taken place in 2010 in the USA [1]. Drunk drivers are involved in approximately one-third of motor vehicle related deaths in the USA and other countries [2]–[4]. The severe consequences of DWI have led the US Center for Disease Control to recommend ways to reduce drunk driving [2]. These suggestions range from individuals taking more responsibility to curb their binge drinking, to implementing new laws and better enforcing existing laws. Altering the availability of alcohol is also known to affect rates of driving after drinking and DWI charges [5]–[7]. For instance, when laws allowing alcohol sales on Sunday were introduced in New Mexico, the number of alcohol-related traffic deaths and crash fatalities increased relative to the pre-allowance period [5]. Although there are some exceptions to this pattern [8], existing data are broadly consistent with the *alcohol availability hypothesis* that higher alcohol accessibility predicts both greater alcohol consumption and consumption-related harm [9]. One method of examining the influence of alcohol availability on DWI charges is by investigating the business hours of social premises licensed to serve alcohol (e.g., pubs, bars, clubs and restaurants). When business hours are longer, alcohol is more available than when hours are shorter.

Previous epidemiological research on alcohol availability has not distinguished between first-time and repeat DWI offending. Determining whether alcohol availability is a risk factor for an increased incidence of first-time drunk driving as well as habitual drunk driving is critical if DWI interventions are to be appropriately selected to match a community’s problems and needs. The present research addressed this gap in knowledge by examining the relationship between alcohol availability and DWI charges in New York State (NY). NY was selected for examination because it is the only American state without dry counties with long-established by-county variation in business hours for alcohol outlets and publically accessible DWI arrest data. In NY a misdemeanor charge applies to the first drunk driving offense with blood alcohol content over.08 [10]. When blood alcohol content is under.08 but driving ability is impaired, a traffic offense of driving while ability impaired may be given. However, it should be noted that a third driving while ability impaired traffic offense in 10 years becomes a misdemeanor charge. Any subsequent drunk driving offense within 10 years necessitates a felony charge. Rates of misdemeanor and felony DWI charges are published publically [11], and were therefore used in the present research. Rates of the driving while ability impaired traffic offense are not published publically.

In the present study, we examined the relationship between alcohol outlet business hours and both misdemeanor and felony DWI charges after controlling for relevant covariates. Moreover, past research has demonstrated that longer outlet business hours in one region affect alcohol-involved motor vehicle incidents in adjacent regions [12]. This likely reflects the fact that people often travel to adjacent regions for the purposes of drinking, and then return through both the region where they were drinking and their home region. Thus, in the present research we also examined the effects of any additional outlet business hours in adjacent counties. It is important to examine whether alcohol outlet business hours contribute equally to first-time and repeat drunk driving. This knowledge can be applied to specifically target and develop interventions for these different types of drunk-drivers. Policy makers, chambers of commerce, and alcohol outlet business owners could also conceivably make use of this information to reduce harm associated with bars and restaurants.

We hypothesized that alcohol availability would increase misdemeanor-level DWI charges. This prediction is consistent with the alcohol availability hypothesis. By contrast, other more individual-level factors may be stronger determinants of felony-level DWI charges than outlet business hours. For instance, people with multiple recorded DWI offenses (i.e., felony-level offending in NY) typically have deficits in executive functioning and higher rates of psychopathology [13]. Beyond this general pattern of risk factors, repeat offenders are also more likely to be involved in traffic accidents while sober and to be arrested for other reasons [13]. Overall repeat offenders appear to be at a high risk of many disinhibited behaviors that first time offenders are not. Thus, we expected a null relationship between outlet business hours and felony-level drunk driving charges.

## Methods

This study utilized publically available data on DWI charges from the counties of NY (*N* = 62). All collated data sets have been archived using WebCite® to ensure their continued availability (URLs of both the original and archived data are provided in the reference list). The hypothesized effects were then reanalyzed for robustness with the 5 counties of New York City (NYC) excluded due to the abnormally low rates of household vehicle availability in NYC (i.e., Bronx, Brooklyn, New York, Queens and Richmond) (*M = *50.63%, *SD* = 23.75) relative to the rest of the state (*M = *91.41%, *SD* = 2.49), *t*(4.01) = 3.84, *p* = .018, *d* = 3.84 [14]. Low rates of vehicle ownership should reduce the propensity to engage in any driving offense.

This study utilized data from 2009. However, in the case of three variables – racial composition, gender composition, and age-structure – data from 2009 were unavailable. As such, data for 2009 were estimated based on census data for the years 2000 and 2010 using the following formula: *Estimate_2009_ =  Census_2010_ - (Census_2010_ - Census_2000_)/10*.

### Independent Variable

#### Outlet business hours

Data on outlet business hours were obtained from the NY Liquor Authority [15]. All counties allow alcohol outlets to sell alcohol for on-premises consumption from 8 a.m. each day. As such, the variation in allowed outlet business hours between counties is due to variation in the cease of sale time. These range between 1 a.m. and 4 a.m. each day, and vary within a county depending on the day of the week. After the cessation of alcohol sales, alcohol may be consumed for an additional 30 minutes [16]. Outlet business hours were summarized as the hours open after midnight each week in each county. The number of hours open after midnight each week ranged from 7 to 28; 7 hours (*n = *5), 8 hours (*n = *1), 9 hours (*n = *3), 11 hours (*n = *2), 13 hours (*n = *1), 14 hours (*n = *19), 15 hours (*n = *1), 21 hours (*n = *3), 27 hours (*n = *1), 28 hours (*n = *24).

#### Adjacent county hours

The additional outlet business hours available in adjacent counties were calculated by taking the maximum business hours of border sharing counties and subtracting from this number the counties’ outlet business hours. The number of additional outlet business hours available in adjacent counties ranged between 0 and 19 hours per night; 0 hours (*n = *38), 1 hour (*n = *1), 2 hours (*n = *3), 5 hours (*n = *2), 6 hours (*n = *1), 7 hours (*n = *6), 10 hours (*n = *2), 12 hours (*n = *1), 14 hours (*n = *6), 15 hours (*n = *1), 19 hours (*n = *1).

### Dependent Variables

#### DWI offenses

The number of DWI arrests in each county was obtained from the NY Division of Criminal Justice Services [11]. Utilizing the population estimates used by the NY Division of Criminal Justice Services in other publications [17], the rate of DWI offenses per 100,000 inhabitants in 2009 was calculated for each county. DWI offenses are broken down into misdemeanor-level (first offense) (*M = *395.57, *SD = *142.51), and felony-level (repeat offense) charges (*M = *65.61, *SD = *30.92).Covariates

#### Per capita alcohol outlets

Alcohol may be sold by a number of different types of business in NY. We calculated the number of businesses per 10,000 people of each major type: on-premises [18] (*M = *2.28, *SD = *0.97), off-premises [19] (*M = *1.27, *SD = *0.63), and restaurants [20] (*M = *9.85, *SD = *4.92). Alcohol may be sold to be consumed on-premises in establishments such as bars, taverns and nightclubs. Alcohol may be sold packaged for off-premises consumption as in a liquor store. Businesses with a license for both on-premises and off-premises consumption are classified as having an on-premises license. Alcohol may also be sold for consumption on-premises by establishments where the consumption of alcohol is not the primary purpose of the business, such as in full-service restaurants.

#### Unemployment and per capita income

At a societal level, levels of stress and hardship may be indexed through markers such as unemployment rates [21] (*M = *8.38, *SD = *1.03) and per capita income [22], [23] (in $1,000 intervals) (*M = *36.98, *SD = *11.92). Average monthly unemployment across 2009 was used as an index of unemployment, and per capita income data for 2009 was as assessed by the U.S. Bureau of Economic Analysis Regional Economic Information System. It is important to control for these factors as stressful circumstances could promote drinking behavior.

#### Population density

Population density was represented by the number of people per square mile of county land area [25]. These values had a positively skewed distribution and so were log_10_ transformed.

#### Racial, gender, and age composition

As being White has been linked to increased drunk driving behavior, we controlled for the proportion of White people in the population [26]. US Census data was used to estimate the proportion of White individuals in each county [27], [28] (racial composition) (*M = *86.38, *SD = *14.04). Societal gender and age composition were also important to control for as drunk driving is typically linked to younger males [26]. To this end, US Census data was used to estimate the number of males per 100 females in the population [27], [28] (gender composition) (*M = *98.69, *SD = *6.06); and the proportion of adults aged 18 through 34 [29], [30] (age composition) (*M = *21.81, *SD = *4.08).

### Statistical Analyses

Separate linear regression analyses were used to test if outlet business hours and additional outlet business hours in adjacent counties predicted rates of misdemeanor-level and felony-level DWI charges. In these analyses, linear effects of population density (log_10_ transformed), per capita income, unemployment, racial composition, gender composition, age structure, and the per capita prevalence of three types alcohol outlets were controlled (i.e., on-premises with alcohol as primary purpose, on-premises with meals as primary purpose, off-premises only). Bivariate correlations between the predictors are presented in Table 1.

**Table 1:** Bivariate correlations between predictor variables.

Variable		(1)	(2)	(3)	(4)	(5)	(6)	(7)	(8)	(9)	(10)	(11)
(1)	Business Hours	1	−.50**	.48**	.40**	−.06	−.48**	−.14	−.06	.09	−.48**	.27*
(2)	Adjacent Business Hours		1	−.37**	−.24	.03	.30*	.20	−.06	.13	.36**	−.21
(3)	Population Density			1	.59**	−.01	−.86**	−.61**	.39**	−.25*	−.42**	.02
(4)	Per Capita Income				1	−.35**	−.41**	−.38**	.15	.31*	−.16	.17
(5)	Unemployment					1	.14	.08	.06	−.16	.13	−.23
(6)	Proportion White						1	.43**	−.40**	.17	.44**	.03
(7)	Gender Composition							1	−.17	.04	.07	−.01
(8)	Younger Age Structure								1	−.17	.12	−.08
(9)	Restaurants									1	.37**	.17
(10)	On-premises outlet										1	−.25*
(11)	Off-premises outlet											1

*Note.* Population Density and Proportion White were strongly negatively correlated. In our analyses using either variable individually, rather than both simultaneously, did not substantially change the core findings pertaining to outlet business hours.

* *p*<.05;

** *p*<.01.

The differential strength of the effect of each independent variable (e.g., outlet business hours) on misdemeanor-level and felony-level DWI rates was extracted by calculating the partial correlation between each independent variable and DWI offenses, controlling for all other variables. Furthermore, the partial correlation between misdemeanor-level and felony-level DWI charges was calculated. With this information, the differential strength of the effect of each factor in the model on misdemeanor-level and felony-level DWI rates may be determined using Steiger’s formula [31]. This formula compares correlation-based effects drawn from the same sample, allowing cross model comparison of effect sizes suitable to the present design. We used DeCoster and Iselin’s [32] Microsoft Excel implementation of the formula.

## Results

Analysis of the full regression models is presented in Table 2. A significant positive relationship was identified between outlet business hours and misdemeanor-level DWI charges, but not with felony-level DWI charges. Every 1 hour increase in weekly outlet business hours was significantly associated with 5.78 more misdemeanor DWI charges per 100,000 people (95% CI: 1.52, 10.04; *t*(50) = 2.72, *p = *.009), while every extra hour available in an adjacent county was significantly associated with 5.18 more misdemeanor DWI charges per 100,000 people (95% CI: 0.00, 10.37; *t*(50) = 2.01, *p = *.050). In contrast, weekly outlet business hours were not associated with felony DWI charges (95% CI: −1.35, 0.66; *t*(50) = 0.68, *p = *.497), nor were hours in adjacent counties (95% CI: −0.69, 1.76; *t*(50) = 0.88, *p = *.385). Outlet business hours were more strongly associated with elevated rates of misdemeanor-level DWI charges than felony-level DWI charges (*z = *3.23, *p = *.001); whereas the effect of additional outlet hours in adjacent counties did not differentially predict misdemeanor and felony level DWI charges (*z = *1.12, *p = *.264). These findings did not change if the counties of NYC were excluded from analysis.

**Table 2:** Regression models of alcohol availability on driving while intoxicated in NYS in 2009.

		Misdemeanor DWI			Felony DWI		
*Mean (SD)*		395.57 (142.51)			65.61 (30.92)		
Variable		*b*	*SE b*	*β*	*b*	*SE b*	*β*
Business Hours	(hours after midnight)	5.78	2.12	0.32**>>	−0.34	0.50	−0.09
Adjacent Business Hours	(hours after midnight)	5.18	2.58	0.20*	0.54	0.61	0.09
Population Density	(Log_10_ people per sq mi)	−34.09	46.09	−0.20	−7.46	10.90	−0.20
Per Capita Income	(/$1000)	−2.36	1.73	−0.20	−0.54	0.41	−0.21
Unemployment	(%)	9.74	13.21	0.07	0.83	3.13	0.03
Proportion White	(% White)	4.90	2.02	0.48*	0.32	0.48	0.15
Proportion Male	(males per 100 females)	1.29	2.66	0.06	0.48	0.63	0.09
Younger Age Structure	(% aged 18 to 34)	3.65	3.57	0.10>	−1.29	0.84	−0.17
Restaurants	(per 10000 people)	5.89	3.73	0.20	0.81	0.88	0.13
On-premises outlet	(per 10000 people)	13.36	18.25	0.09	2.98	4.32	0.09
Off-premises outlet	(per 10000 people)	24.89	20.51	0.11	13.36	4.85	0.27**

*Note.* In the misdemeanor DWI model *R* ^2^ = .68 (*p*<.001), and there were no outliers; in the felony DWI model *R* ^2^ = .62 (*p*<.001), and there were no outliers.

* *p*<.05 in the regression;

** *p*<.01 in the regression.

>indicates that the effect of the variable is more positive for misdemeanor DWI than felony DWI at *p*<.05;

>>indicates that the effect of the variable is more positive for misdemeanor DWI than felony DWI at *p*<.01.

Three other significant patterns emerged in the data. A greater proportion of white people in a county was associated with an increased incidence of misdemeanor-level DWI charges (*t*(50) = 2.43, *p = *.019), and a greater per capita density of off-premises outlets was associated with increased incidence of felony-level DWI charges (*t*(50) = 2.75, *p = *.008). Finally although county age structure was unrelated to rates of either DWI offense, it was a significant predictor of DWI offense severity. Specifically, counties with younger age structures compared to those with older age structures tended to experience a higher proportion of misdemeanor-level charges relative to felony-level charges.

When the counties of NYC were excluded from analysis, per capita restaurants were additionally positively correlated with misdemeanor-level DWI charges (*t*(45) = 2.22, *p = *.031), and per capita income was negatively correlated with felony-level DWI charges (*t*(45) = 2.12, *p = *.040). Similar patterns occur if population density, which is both highly correlated with the proportion of White people in a county and distinguishes the NYC counties from non-NYC counties, is dropped from the regression model. Specifically, per capita restaurants were positively correlated with misdemeanor-level DWI charges (*t*(51) = 2.40, *p* = .020) and per capita income was negatively correlated with misdemeanor-level DWI charges (*t*(51) = 2.39, *p* = .021). Per capita income was negatively correlated with felony-level DWI charges, *t*(51) = 2.23, *p = *.026. However, the same cannot be said when the proportion of White people is dropped from the regression model – suggesting that population density may be obscuring these effects.

We conducted three separate post hoc analyses using forward regression to examine possible interactions and quadratic effects. We determined entry criteria using the False Discovery Rate technique [33] to conservatively account for the sheer volume of possible exploratory effects. These analyses indicated that outlet business hours did not interact with any of the covariates, the covariates did not interact with each other, and that the linear effect of outlet business hours on misdemeanor-level DWI was not significantly qualified by a quadratic relationship.

Post hoc, we additionally examined the effects of controlling for relationship status, which may be another social factor related to drinking behavior. Marital stress may promote drinking, while singles may go out drinking more than married people. We explored potential effects of marital status [24] on offending. It should be noted that the proportion of never married individuals was correlated highly with the proportion of individuals aged 18 to 34 (*r* = .87). As such, we focused on the alternate possibility that relationship stress may promote drinking. We calculated the ratio of married individuals to separated and divorced individuals for each county, and calculated an additional ratio including widowed individuals. These variables were unrelated to either misdemeanor-level or felony-level DWI, and did not change the patterns for other variables.

## Discussion

In this cross-sectional study of NY, longer business hours for alcohol-serving establishments, including those additional hours available in adjacent counties, were significantly associated with higher rates of misdemeanor-level DWI charges. Business hours were not significantly associated with population rates of felony-level DWI charges. The pattern of results, and a comparison of partial correlation coefficient magnitudes, supported our hypotheses that alcohol availability would positively correlate with first-time but not repeat DWI offenses.

Although the present study is correlational, prior research does support a causal role of alcohol availability on DWI rates [5]–[7]. The present research extends prior work [5]–[7] by showing that the temporal availability of alcohol was positively associated with rates of first-time offending, but not repeat offending. These findings suggest that alcohol availability may be an impelling factor for drunk driving, but is unlikely to maintain drunk driving behaviors. Indeed, in light of research on repeat DWI perpetrators [13], maintaining factors are likely to be those affecting the individual (e.g., psychopathology, tendency toward impulsivity), rather than those affecting society more broadly (e.g., alcohol availability, punitive sanctions).

Given the correlational design, we cannot make firm causal conclusions about the influence of outlet business hours on drunk driving. For instance, it is possible that those who tend to get arrested for drunk driving gravitate toward counties with later business hours. Indeed the finding that adjacent county hours were correlated with first-time DWI arrests suggests that people may drive to nearby counties to drink later and subsequently get arrested near to home [12]. Alternatively, outlet business hours may have been extended due to the same societal attitudes which promote drunk driving. However, as allowed alcohol outlet business hours have not changed in recent times, this explanation is less likely than the former. Short of intervention designs, stronger evidence for a causal relationship between outlet business hours and DWI offenses may be obtained in future correlational research by examining the time of offense and the location of alcohol consumption. Examining these factors will determine the extent to which drunk driving occurs in the period of, or just after, any additional temporal alcohol availability while being able to concretely link consumption to a premises.

One possible explanation for the null relationship between business hours and felony arrests may be due to punitive measures that reduce repeat offending. This study utilized data from 2009. On December 18, 2009 the Child Passenger Protection Act (Leandra’s Law) came into effect [34], making it a requirement to fit an ignition interlock device after any DWI conviction, and making any DWI charge a felony if a child is in the vehicle. However, the implementation of this law is unlikely to have had significant effects on the present data, as it was only in effect for 13 days of 2009. In the period prior to this law, DWI convictions carried a fine and the possibility of up to one year jail time. Additionally, if convicted of a misdemeanor charge, a person’s license would typically be revoked for 6 months (12 months for felony convictions) [35], a consequence that could be overturned if the perpetrator completed a rehabilitation program [36]. Furthermore, limited privilege conditional licenses could be applied for, which allow the perpetrator to drive between home and work only.

In the present study there was no effect of the spatial availability of on-premises alcohol serving establishments on drunk driving. Although past studies reported positive associations, they have either relied on crash and injury data [37], [38] or on self-reported drunk driving [39]–[41]. When outlet density is high, despite alcohol being more available, people do not need to drive as far to obtain it. This subsequently reduces the amount of time they will spend driving intoxicated and therefore the likelihood that they will be arrested for drunk driving (the dependent measure in the present study) even if they would self-report doing so.

There were additional significant relationships between the control variables and arrest rates in the present study. The fact that younger age was associated with relatively more misdemeanors and less felonies is consistent with the fact that younger people have had less time to reoffend. However, these effects are inconsistent with a study of first-time and repeat DWI offenders in Mississippi and Colorado who were referred by the courts to treatment programs and community service [13]. In that study, there was no age difference between first-time and repeat offenders. The finding that a greater proportion of White residents in a county predicted greater misdemeanor-level DWI offenses is consistent with past research on racial differences in drunk driving behavior using self-report methods [26], [42]. Finally, the finding that rates of felony DWI charges were associated with greater off-premises outlet density suggests that it is not only the temporal availability of alcohol which affects DWI rates, but also the spatial availability of alcohol for purchase and later consumption.

Calculations that only one in eighty instances of self-reported drunk driving in the US are caught by police should temper interpretation of the present results [43]. Because it is unlikely to be caught, it is possible that the positive correlation between outlet business hours and misdemeanor DWI is unique to the type of DWI events that police detect. Furthermore, assuming that the present effects prove to be broadly generalizable, our findings suggest a number of policy implications. First, altering the temporal availability of alcohol is unlikely to prevent repeat offending. Due to the likely presence of individual-level maintaining factors for repeat offenders, court-ordered interventions and sanctions may be more appropriate for this population. In contrast, reducing the temporal availability of alcohol may reduce the likelihood that the non-DWI convicted, young, White population will drive drunk.

The present findings also have implications when planning the geographic scope of an intervention. Single-county reductions in the temporal availability of alcohol are unlikely to be as effective as more widespread reductions (multiple-county, or state-wide), due to the effects of adjacent county hours in the present study. Moreover, because the relationship between outlet business hours and first-time drunk driving was not moderated by population density, reducing outlet business hours should theoretically reduce first-time drunk driving in both rural and suburban environments. This means that widespread interventions are likely to work uniformly across region-type. However, because of the uniform outlet business hours in NYC, we were unable to determine whether reducing outlet business hours would reduce drunk driving *within* major cities.

In conclusion, longer hours of alcohol service were positively associated with higher rates of DWI misdemeanors, but not felonies. To our knowledge, this is the first study to separate the effects of outlet business hours on first-time and repeat DWI offenses. The unique effects of outlet business hours on first-time, or misdemeanor-level, DWI offenses may be informative for the development of interventions and policies. Specifically, societal level changes in hours of alcohol service may reduce first-time DWI offending. A future step may be to evaluate whether outlet business hour interventions would be limited to preventing first-time DWI offenses, as is suggested by our results. Furthermore, as temporal alcohol availability was unrelated to felony-level charges, consideration should be given to developing and testing special interventions for repeat DWI offenders.

The present research adds to a growing body of literature documenting the relationship between the temporal availability of alcohol and harm. This harm goes beyond drunk driving and the accidents it causes, encompassing public violence and the damage it causes to both the individual and society [44]–[48]. Reducing outlet business hours can reduce other forms of alcohol related harm, such as drunken assaults [45], [46]. Thus, serious consideration should be given to whether similar interventions can also curb first-time drunk driving offenses.
